# Genome-level comparisons provide insight into the phylogeny and metabolic diversity of species within the genus *Lactococcus*

**DOI:** 10.1186/s12866-017-1120-5

**Published:** 2017-11-03

**Authors:** Jie Yu, Yuqin Song, Yan Ren, Yanting Qing, Wenjun Liu, Zhihong Sun

**Affiliations:** Key Laboratory of Dairy Biotechnology and Engineering, Ministry of Education, Key Laboratory of Dairy Products Processing, Ministry of Agriculture, Inner Mongolia Agricultural University, Hohhot, China

**Keywords:** *Lactococcus*, Comparative genomics, Carbohydrate metabolism, Phylogeny

## Abstract

**Background:**

The genomic diversity of different species within the genus *Lactococcus* and the relationships between genomic differentiation and environmental factors remain unclear. In this study, type isolates of ten *Lactococcus* species/subspecies were sequenced to assess their genomic characteristics, metabolic diversity, and phylogenetic relationships.

**Results:**

The total genome sizes varied between 1.99 (*Lactococcus plantarum*) and 2.46 megabases (Mb; *L. lactis* subsp*. lactis*), and the G + C content ranged from 34.81 (*L. lactis* subsp*. hordniae*) to 39.67% (*L. raffinolactis*) with an average value of 37.02%. Analysis of genome dynamics indicated that the genus *Lactococcus* has an open pan-genome, while the core genome size decreased with sequential addition at the genus and species group levels. A phylogenetic dendrogram based on the concatenated amino acid sequences of 643 core genes was largely consistent with the phylogenetic tree obtained by 16S ribosomal RNA (rRNA) genes, but it provided a more robust phylogenetic resolution than the 16S rRNA gene-based analysis.

**Conclusions:**

Comparative genomics indicated that species in the genus *Lactococcus* had high degrees of diversity in genome size, gene content, and carbohydrate metabolism. This may be important for the specific adaptations that allow different *Lactococcus* species to survive in different environments. These results provide a quantitative basis for understanding the genomic and metabolic diversity within the genus *Lactococcus*, laying the foundation for future studies on taxonomy and functional genomics.

**Electronic supplementary material:**

The online version of this article (10.1186/s12866-017-1120-5) contains supplementary material, which is available to authorized users.

## Background

*Lactococcus* is a genus of catalase-negative, gram-positive, non-motile, facultative anaerobic bacteria that typically inhabit animals, plants, and their related products, particularly fermented products; they are generally considered nonpathogenic toward humans [1]. *Lactococcus* species have variable and abundant nutritional requirements. They usually grow within a temperature range of 10–40 °C, although some species are capable of growing at temperatures as low as 7 °C over a prolonged incubation period of 10–14 days [2]. Most *Lactococcus* species can grow in 4.0% (*w*/*v*) NaCl; in media, they grow best at pH 7.0 and cease to grow if the pH drops to 4.5 [3].

*Lactococcus lactis* was the first species in the genus to be identified (by Joseph Lister in 1873) and then it was later renamed *Streptococcus lactis* [1]. Schleifer et al. defined the genus *Lactococcus* as distinct from the genus *Streptococcus* [4]. To date, there are 12 recognized species with four further subspecies in the genus (http://www.bacterio.net/lactococcus.html, December 2016). Some species, especially *L*. *lactis*, are important in the manufacturing of fermented milk products and some species produce antimicrobial compounds, such as bacteriocins, nisin, and lactococcin [5, 6], and recombinant proteins [7]. Some probiotic foods contain these species, and there have been more studies regarding the relationships between these probiotic-containing functional foods and the maintenance of human intestinal health [8, 9]. One species, *Lactococcus garvieae*, is a major pathogen of fish causing fatal hemorrhagic septicemia [10]. However, *L*. *garvieae* has also been reported as a common component of fermented dairy manufactured from raw milk [11–14], and phenotypically it is closely related to *L*. *lactis*.

The number of studies using high-throughput sequencing to study bacterial metabolism, evolution, and speciation within genera has increased markedly with the advances and decreasing costs of sequencing technologies [15–17]. Whole genomic sequences allow reconstruction of reliable phylogenies based on much larger sequence data sets than previously available. This makes it possible to gain an understanding of the genomic diversification of entire genera in great detail [18]. The first genome of a lactic acid bacterium (LAB), *L. lactis* isolate IL1403, was sequenced in 2001 [19]. Subsequently, there have been increasing reports on whole genome sequences of LAB. However, of the 16 currently recognized species/subspecies in the genus *Lactococcus*, only seven have been fully sequenced (National Center for Biotechnology information [NCBI]): *L*. *lactis* subsp*. lactis*, *L*. *lactis* subsp*. cremoris*, *L*. *garvieae*, *L. raffinolactis*, *L. plantarum*, *L. fujiensis*, and *L. piscium* [20–23]. With many new species being identified in the genus *Lactococcus*, a genomic view could provide important information to understand bacterial evolution and adaptation to different environments. Moreover, comparative genomic of species within the same genus can reveal the genomic diversity within a genus and provide insights into how environmental conditions affect the evolution of different species.

In the present study, the genomes of ten type species/subspecies in the genus *Lactococcus* were sequenced. We then used comparative genomic approaches to define the pan-genome, core genome, and unique genes; assess genetic diversity and species taxonomy; infer phylogenetic relationships; and study the mechanisms of carbohydrate metabolism.

## Methods

### Bacterial isolates, DNA extraction and carbohydrate utilization

The ten type isolates of *Lactococcus* species/subspecies, as defined by the LPSN (http://www.bacterio.net/) were collected from the German Collection of Microorganisms and Cell Cultures (DSMZ; Table 1). These isolates were cultured in M17 broth (Oxoid Ltd., Basingstoke, UK) at 37 °C for 24 h.

**Table 1:** List of the isolates used in the evaluation of the genus *Lactococcus*

Type strains	Species	Source	Isolation Year	References
DSM 22330	*Lactococcus chungangensis*	activated sludge foam	2008	[46]
JCM 16395	*Lactococcus fujiensis*	vegetable matter	2011	[47]
ATCC 19257	*Lactococcus lactis* subsp*. cremoris*	cream	1919	[4]
DSM 20450	*Lactococcus lactis* subsp*. hordniae*	leaf hopper	1977	[4]
ATCC 19435	*Lactococcus lactis* subsp*. lactis*	milk	1873	[4]
DSM 21502	*Lactococcus lactis* subsp*. tructae*	intestinal mucus of brown trout (*Salmo trutta*) and rainbow trout	2011	[35]
DSM 6634	*Lactococcus piscium*	salmonid fish	1990	[48]
DSM 20686	*Lactococcus plantarum*	frozen peas	1984	[4]
ATCC 43920	*Lactococcus raffinolactis*	milk	1932	[4]
DSM 20684	*Lactococcus garvieae*	bovine mastitis	1984	[4]

Bacterial DNA extraction kits (OMEGA D3350–02) were used for DNA extraction from each isolate according to the manufacturer’s instructions. Quantitative and qualitative analysis of genomic DNA was achieved using electrophoresis on 1% agarose gels and a TBS-380 mini fluorometer (Turner BioSystems Inc., Sunnyvale, CA). Only good quality DNA samples (OD260/280 = 1.8~2.0, >6μg) were used to construct fragment libraries (200 to 300 bp).

Carbohydrates fermentation was tested using the API 50 CH systems (Biomerieux, France) based on the manufacturer’s instructions.

### Genome sequencing, assembly, prediction and annotation of coding sequences (CDS), and pathway mapping

The ten genomes of the the *Lactococcus* type species/subspecies were sequenced using an Illumina HiSeq 2000 (Illumina Inc. USA) by generating paired-end libraries. The average length was 300 bp, and 838 Mb of high quality data was generated for each isolate consistent with a sequencing depth of 129- to 388-fold. Assembly of the paired-end reads was performed using SOAPdenovo v2 [24]. The software Gap Closer was used to fill the local inner gaps and correct single base errors (http://sourceforge.net/projects/soapdenovo2/files/GapCloser/). Gene prediction and functional annotation of predicted genes were achieved using RAST 2.0 [25, 26]. The individual genome assemblies were deposited in the NCBI, the accession numbers are shown in Table 2. Carbohydrate metabolism related pathway information was identified from the pathways described in the Kyoto Encyclopedia of Genes and Genomes (KEGG, http://www.kegg.jp/) [27].

### Core genome, pan-genome and unique gene analysis

For predicting the possible dynamic changes in genome size, the sizes of the core genome (common genes, mutually conserved), pan-genome (gene repertoire) and unique genes (specific genes, only found in one genome) were calculated. Owing to large variation were happened in the homologous genes between species within a genus, the concept of ‘gene family’ was used to take the place of the generally used term ‘gene’. According to previous research [28, 29], the core genome, pan-genome and unique genes [30] of *Lactococcus* were estimated. A pair of genes was placed in the same gene family, when their amino acid sequences identity was >50%, and when more than 50% of the amino acid sequences of the longer gene could be covered by the shorter one.

For construction of the pan-genome, all of the predicted genes were first grouped into possible gene families for each genome and then the gene families of ten genomes were accumulated. The core genome was built by counting the number of commonly shared gene families within ten genomes, while the unique gene family was defined as the gene families that could only be found in one genome.

### The average nucleotide identity (ANI) and phylogenetic analysis

The pair-wise ANI values of ten *Lactococcus* species/ subspecies were calculated using the methods of Goris et al. [30]. To infer phylogenetic relationships among *Lactococcus* species, we constructed a maximum likelihood (ML) tree based on the concatenated amino acid sequences of 643 core genes. *Streptococcus* (*S.*) *thermophilus* LMG 18311 was used as an out-group because it is phylogenetically closely related to the genus *Lactococcus* [31]. Amino acid sequences of the core genes were aligned by MUSCLE v3.8.31 [32], and the software PHYML was used to construct the ML tree with the WAG model and 500 bootstrap iterations [33]. In addition, a Neighbour-Joining (NJ) tree was inferred based on 16S rRNA sequences downloaded from Genbank for all ten type species/subspecies of *Lactococcus* using Mega 6.0 [34] (http://www.megasoftware.net) with 1000 bootstrap iterations; again *S. thermophilus* LMG 18311 was used as an out-group.

## Results

### General features

The estimated genomes of the type isolates of ten *Lactococcus* species/subspecies were sequenced and assembled into 43–226 scaffolds. Across species, the total genome size varied from 1.99 (*L*. *plantarum*) to 2.46 megabases (Mb; *L*. *lactis* subsp*. lactis*), with a mean value of 2.27 Mb (Table 2). In addition, the G + C content ranged from 34.81 (*L*. *lactis* subsp*. hordniae*) to 39.67% (*L*. *raffinolactis*) with an average value of 37.02% (Table 2). The numbers of predicted coding genes varied from 1861 (*L*. *plantarum*) to 2684 (*L*. *lactis* subsp*. hordniae*).

**Table 2:** Genomic features of species/ subspecies in the genus *Lactococcus*

Type strains	Scaffold number	Genome size (Mb)	GC%	Predicated coding genes	Genbank accession number
*L. chungangensis* DSM 22330^a^	86	2.20	38.67	2154	JXJT00000000
*L. garvieae* DSM 20684	71	2.02	38.52	1995	JXJV00000000
*L. fujiensis* JCM 16395	69	2.08	36.95	2057	JXJU00000000
*L. lactis* subsp. *cremoris* ATCC 19257	226	2.32	35.55	2477	JXJZ00000000
*L. lactis* subsp. *hordniae* DSM 20450	101	2.43	34.81	2684	JXKA00000000
*L. lactis* subsp. *lactis* ATCC 19435	76	2.46	35.23	2500	JXKB00000000
*L. lactis* subsp. *tructae* DSM 21502	61	2.61	35.49	2612	JXKC00000000
*L. piscium* DSM 6634	74	2.41	38.55	2301	JXJW00000000
*L. plantarum* DSM 20686	43	1.99	36.76	1861	JXJX00000000
*L. raffinolactis* ATCC 43920	121	2.21	39.67	2143	JXJY00000000

^a^ *L. Lactococcus*

### Pan-genome and core genome analysis

**Fig. 1:**
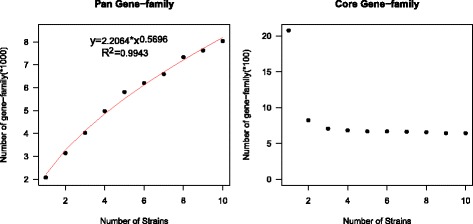
Pan-genome and core genomes of species/subspecies in the *Lactococcus* genus. The gene accumulation curves show the number of new genes (pan-genome) and genes in common (core-genome) obtained by adding a new genome to a previous set. The pan-genome curve is the least squares fit of the power law to these values

The pan-genome, defined as the full complement of genes from the ten *Lactococcus* genomes evaluated, contained 8036 gene families and grew continuously as each genome was added; a mean of 662 gene families were added for each genome (Fig. 1a). Compare to the pan-gene families, the amount of core gene families decreased sharply along with the number of genomes increased, reaching a minimum value of 643 for all ten genomes (Fig. 1b). The proportion of core genes varied between 23.9 (*L*. *plantarum*) and 34.6% (*L*. *lactis* subsp*. hordniae*) of the total predicted coding genes for the ten *Lactococcus* species/subspecies. These observations indicated a high degree of genomic diversity among the ten type species/subspecies of the genus *Lactococcus*. Such genome diversity in the genus *Lactococcus* is not surprising considering the range of dairy, plant, and animal environments from which the species were isolated.

**Fig. 2:**
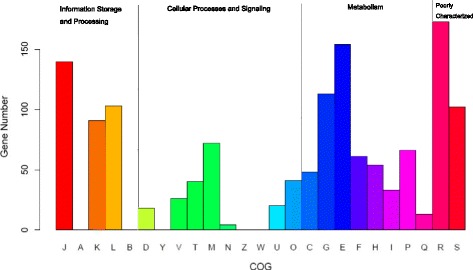
Distribution of functional categories of core gene families in the *Lactococcus* genus. Cluster of Orthologous Group (COG) functional classification description — Information Storage and Processing: [A] RNA processing and modification; [B] chromatin structure and dynamics; [J] translation, ribosomal structure, and biogenesis; [K] transcription; [L] replication, recombination, and repair. Cellular Processes and Signaling: [D] cell cycle control, cell division, chromosome partitioning; [M] cell wall/membrane/envelope biogenesis; [N] cell motility; [O] posttranslational modification, protein turnover, chaperones; [T] signal transduction mechanisms; [U] intracellular trafficking, secretion, and vesicular transport; [V] defense mechanisms; [W] extracellular structures; [Y] nuclear structure; [Z] cytoskeleton. Metabolism: [C] energy production and conversion; [E] amino acid transport and metabolism; [F] nucleotide transport and metabolism; [H] coenzyme transport and metabolism; [G] carbohydrate transport and metabolism; [I] lipid transport and metabolism; [P] inorganic ion transport and metabolism; [Q] secondary metabolites biosynthesis, transport, and catabolism. Poorly Characterized: [R] general function prediction only; [S] function unknown

To examine the functional characteristics of the core genes, the Cluster of Orthologous Group (COG) database was used to investigate the distribution of core proteins mapped to each COG category (Fig. 2). There were eight subcategories in metabolism, seven subcategories in cellular processes and signaling, three subcategories in information storage and processing and two subcategories that were poorly characterized. The information storage and processing category accounted for 32.3% of the clusters, and the cellular processes and signaling category and metabolism categories accounted for 17.4 and 29.7% of clusters, respectively (Fig. 2). Furthermore, 18.66% of core genes were belong to translation, ribosomal structure, and biogenesis (J); 7.93% were involved in replication, recombination, and repair (L); and 6.22% were involved in cell wall/membrane/envelope biogenesis (M). Other than the functional subcategories J, L, and M, the 17 remaining functional subcategories included 6–38 core genes. It is notable that most of the core genes played important roles in maintaining growth and reproduction in the genus *Lactococcus*; these genes are indispensable and constitute the basic framework of the *Lactococcus* genome.

### Phylogenetic relationships of *Lactococcus* strains

**Fig. 3:**
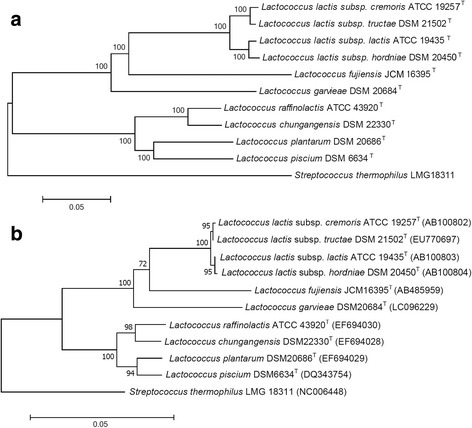
Phylogenetic relationships among species in the *Lactococcus* genus. The trees were constructed with concatenated amino acid sequences of core genes (**a**) and the 16S rRNA gene (**b**) using maximum likelihood and neighbor-joining methods, respectively. *Streptococcus thermophilus* LMG 18311 served as outgroup for both trees

In order to infer phylogenetic position of ten species within the genus *Lactococcus*, phylogenetic trees were constructed using the 16S ribosomal RNA (rRNA) gene and the concatenated amino acid sequences of 643 core genes from the ten type isolates. The ML tree generated a reliable delineation of phylogenetic relationships across the *Lactococcus* species, because of more nodes were supported by 100% of the bootstrap iterations (Fig. 3a). According to this highly robust ML tree, the ten *Lactococcus* species/subspecies were divided into two branches; one branch contained *L*. *raffinolactis* ATCC 43920^T^, *L*. *chungangensis* DSM 22330^T^, *L*. *plantarum* DSM 20686^T^, and *L*. *piscium* DSM 6634^T^, while the other branch included four *L*. *lactis* subspecies, *L*. *fujiensis* JCM 16395^T^, and *L*. *garvieae* DSM20684^T^.

The topologies of the 16S rRNA gene trees were similar to the ML tree (Fig. 3b). However, the resolution and accuracy of the tree of concatenated amino acid sequences was better than that of the 16S rRNA gene tree. For instance, ATCC 19257^T^ and *L*. *lactis* subsp. *tructae* DSM 21502^T^, *L*. *lactis* subsp. *hordniae* DSM 20450^T^ and *L*. *lactis* subsp. *lactis* ATCC19435^T^ were clustered closely in the 16S rRNA gene tree and shared > 99.90% identity, but were divided in the orthologous proteins tree. Both trees proved that the ten isolates clustered together to form a single genus.

### Genetic diversity and species taxonomy

**Fig. 4:**
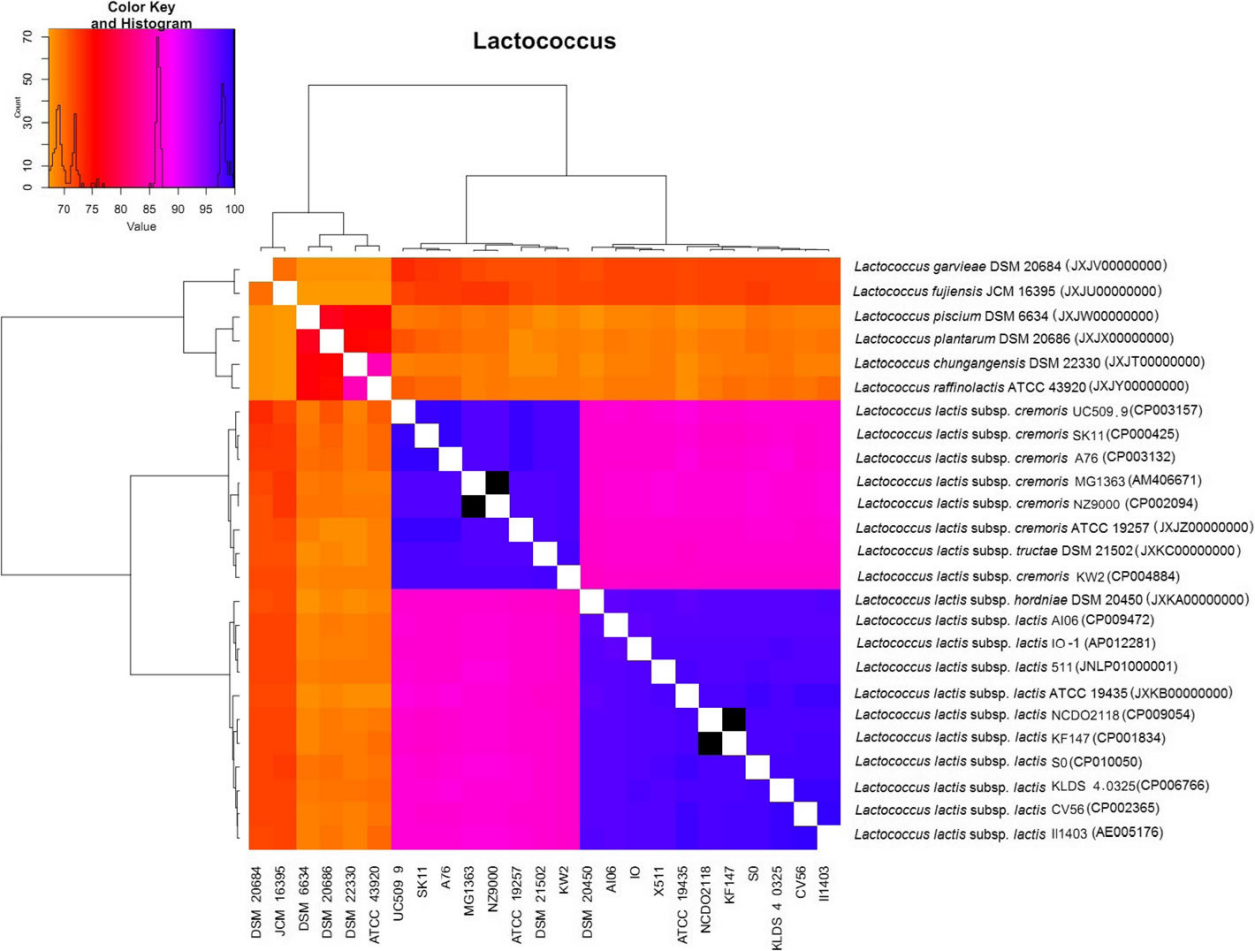
Pairwise average nucleotide identity (ANI) values across ten *Lactococcus* genomes. The colours in the heat map represent pairwise ANI values, with a gradient from yellow (low identity) to blue (high identity)

The ANI value is always used to estimate the genetic distance between isolates, which means the sequence identities of the conserved regions between two genomes [29]. The pair-wise ANI values of ten *Lactococcus* species/ subspecies were calculated to study the interspecies genetic relatedness within the genus *Lactococcus*. Two clusters formed when the ANI for *Lactococcus* species was analyzed (Fig. 4). One cluster contained two subgroups: subgroup I (*L*. *lactis* subsp. *hordniae* and *L*. *lactis* subsp. *lactis*) and subgroup II (*L*. *lactis* subsp. *cremoris* and *L*. *lactis* subsp. *tructae*). The 16S rRNA gene identity for the four subspecies was > 99% [35]; however, the ANI value for this cluster was 86.1–97.9%. It is worth noting that the pairwise ANI values between the two subgroups range from 86.1–86.5%, below the recommended 95% threshold value for species circumscription [36]. To further determine the relationships between the four subspecies, we performed additional comparative genomics with 15 genomic sequences of *L*. *lactis* subsp. *lactis* and *L*. *lactis* subsp. *cremoris* from NCBI. The ANI value distribution of *Lactococcus* is shown in Additional file 1: Table S1, and the heat map was built based on pairwise ANI values (Fig. 4). Based on these results, the four strains were clearly divided into two subgroups, and these values were in line with the DNA–DNA hybridization results. DNA relatedness values between the type strains *L*. *lactis* subsp. *tructae* and *L. lactis* subsp. *hordniae*, *L*. *lactis* subsp. *lactis*, *L*. *lactis* subsp. *cremoris*were 60, 62, and 90%, respectively [35].

Therefore, we suggest that *L*. *lactis* subsp. *cremoris* ATCC 19257^T^ and *L*. *lactis* subsp. *tructae* DSM 21502^T^ should be separated from *L*. *lactis* subsp. *hordniae* DSM 20450^T^ and *L*. *lactis* subsp. *lactis* ATCC 19435^T^ as a single species on the basis of the genetic distance and the phylogenetic relationships shown in ML tree (Fig. 3a). The other cluster contained six type isolates with 16S rRNA gene identity of 91–97% and ANI of 67.4–85.0%. These are the classical delimitation criteria for different species within one genus.

### Carbohydrate metabolism

Carbohydrate metabolism plays a key role in the growth of *Lactococcus* species. On average, 12.5% of the genes in each *Lactococcus* genome are associated with carbohydrate metabolism. Among the core genes of the ten *Lactococcus* species/subspecies evaluated, a total of 38 genes (5.9%) were associated with carbohydrate metabolism. The majority were involved in glycolysis/gluconeogenesis and the pentose phosphate pathway (PPP). Twenty-eight key enzymes that contribute to glycolysis/gluconeogenesis and PPP were encoded (Table 3, Additional file 2: Figure S1). Our results indicated that glycolysis/gluconeogenesis and the PPP are the central carbohydrate metabolism pathways of *Lactococcus* species. In addition, all strains have genes encoding fructokinase (EC 2.7.1.4), phosphoenolpyruvate–protein phosphotransferase (EC 2.7.1.199), and glucose-6-phosphate isomerase (EC 5.3.1.9), which are key enzymes in fructose and mannose metabolism. Thus, all strains could ferment d-glucose, d-fructose, and d-mannose (Table 4). Pathways enabling the use of l-arabinose, l-sorbose, l-rhamnose, xylitol, d-lyxose, and d-tagatose as energy were not found in the genome sequences of the ten strains examined.

**Table 3:** Critical enzymes were coded by core genes of ten *Lactococcus* species/ subspecies for contributing to the glycolysis/ gluconeogenesis and pentose phosphate pathway

No.	EC Number	enzymes
1	EC 1.1.1.1	Alcohol dehydrogenase
2	EC 1.1.1.27	L-lactate dehydrogenase
3	EC 1.1.1.44	6-phosphogluconate dehydrogenase, decarboxylating
4	EC 1.1.1.49	Glucose-6-phosphate 1-dehydrogenase
5	EC 1.2.1.12	glyceraldehyde-3-phosphate dehydrogenase
6	EC 1.2.4.1	pyruvate dehydrogenase E1 component
7	EC 1.2.7.1	Pyruvate-flavodoxin oxidoreductase
8	EC 1.8.1.4	dihydrolipoamide dehydrogenase
9	EC 2.2.1.1	Transketolase
10	EC 2.3.1.12	Dihydrolipoamide acetyltransferase
11	EC 2.7.1.11	6-phosphofructokinase
12	EC 2.7.1.2	Glucokinase
13	EC 2.7.1.40	pyruvate kinase
14	EC 2.7.1.40	Pyruvate kinase
15	EC 2.7.2.3	Phosphoglycerate kinase
16	EC 2.7.6.1	Ribose-phosphate pyrophosphokinase
17	EC 3.1.1.31	6-phosphogluconolactonase
18	EC 3.1.3.11	Fructose-1,6-bisphosphatase
19	EC 3.2.1.86	6-phospho-beta-glucosidase
20	EC 4.1.2.13	Fructose-bisphosphate aldolase class II
21	EC 4.2.1.11	Enolase
22	EC 5.1.3.1	Ribulose-phosphate 3-epimerase
23	EC 5.1.3.3	Aldose 1-epimerase
24	EC 5.3.1.1	Triosephosphate isomerase
25	EC 5.3.1.6	Ribose 5-phosphate isomerase A
26	EC 5.3.1.9	Glucose-6-phosphate isomerase
27	EC 5.4.2.11	2,3-bisphosphoglycerate-dependent phosphoglycerate mutase
28	EC 5.4.2.7	Phosphopentomutase

**Table 4:** Phenotypic characteristics of ten type strains in *Lactococcus* genus

Carbohydrate	DSM 22330	DSM 20684	JCM 16395	ATCC 19257	DSM 20450	ATCC 19435	DSM 21502	DSM 6634	DSM 20686	ATCC 43920
control	–	–	–	–	–	–	–	–	–	–
L-Arabinose	–	–	–	–	–	–	–	–	–	–
Ribose	–	+	+	–	–	+	+	–	–	–
D-xylose	–	–	–	–	–	+	–	+	–	+
D-Glucose	+	+	+	+	+	+	+	+	+	+
D-Fructose	+	+	+	+	+	+	+	+	+	+
D-Mannose	+	+	+	+	+	+	w^a^	+	+	+
L-Sorbose	–	–	–	–	–	–	–	–	–	–
L-Rhamnose	–	–	–	–	–	–	–	–	–	–
D-Sorbitol	–	–	–	–	–	–	–	–	+	–
Amygdalin	+	+	–	–	–	w	+	+	+	–
D-Cellobiose	+	+	+	–	+	+	+	+	+	+
D-Maltose	+	+	+	–	+	+	+	+	+	+
D-Lactose	–	+	+	+	+	+	+	+	–	+
D-Melibiose	–	–	–	–	–	–	+	+	–	+
D-Sucrose	+	–	–	–	+	–	+	+	+	+
D-Trehalose	+	+	+	–	–	+	+	+	+	+
D-Raffinose	–	–	–	–	–	–	+	+	–	+
Xylitol	–	–	–	–	–	–	–	–	–	–
D-Lyxose	–	–	–	–	–	–	–	–	–	–
D-Tagatose	–	–	–	–	–	–	–	–	–	–

^a^ *w* weakly positive

Some differences in carbohydrate metabolism genes were also found among the ten *Lactococcus* species/subspecies. The number of carbohydrate metabolism genes in the ten species/subspecies evaluated varied from 194 (*L*. *plantarum* DSM 20686^T^) to 366 (*L*. *lactis* subsp. *tructae* DSM 21502^T^). Pairwise comparisons among the ten *Lactococcus* species/subspecies showed that the number of strain-specific carbohydrate metabolism genes varied between 2 and 15, and appeared to reflect the functional diversity observed for each isolate. To detect differences in functional genes related to carbohydrate metabolism, acid production from carbohydrates was tested using the API 50 CH system (bioMérieux, Inc., Marcy l’Etoile, France) (Table 4). Raffinose can be enzymatically hydrolyzed to melibiose by β-fructofuranoside (EC 3.2.1.26), and melibiose is further hydrolyzed to d-glucose (α-galactosidase; EC 3.2.1.22), and enters the glycolysis pathway. Three strains, *L*. *lactis* subsp. *tructae* DSM 21502^T^, *L*. *piscium* DSM 6634^T^, and *L*. *raffinolactis* ATCC 43920^T^, can utilize raffinose and melibiose, because they possess the key enzymes (EC 3.2.1.26 and EC 3.2.1.22) for raffinose and melibiose metabolism, while *L*. *plantarum* DSM 20686^T^, *L*. *lactis* subsp. *hordniae* DSM 20450^T^, and *L*. *chungangensis* DSM 22330^T^ with β-fructofuranoside and no α-galactosidase, cannot utilize raffinose and melibiose. Furthermore, β-fructofuranoside is a key enzyme for sucrose hydrolysis, and so the four strains lacking this enzyme cannot utilize sucrose. The other six strains can hydrolyze sucrose to d-glucose-6P and d-fructose. The role of *L*. *lactis* as an important industrial starter strain is mainly due to the rapid conversion of lactose to lactic acid. Lactose is hydrolyzed by β-galactosidase (EC 3.2.1.23) to give α-d-glucose and d-galactose, and it can also be catalyzed to lactose 6-phosphate (Lac-6P) by lactose phosphotransferase (EC 2.7.1.207). Lac-6P is subsequently hydrolyzed by 6-phospho-β-galactosidase (EC 3.2.1.85) to give galactose 6-phosphate (Gal-6P), and Gal-6P is catabolized by the tagatose 6-phosphate (Tag-6P) pathway [37, 38]. Comparative genomic analysis showed that most strains have the key enzymes for lactose metabolism, with the exception of *L*. *chungangensis* DSM 22330^T^, *L*. *fujiensis* JCM16395^T^, *L*. *plantarum* DSM 20686^T^, and *L*. *lactis* subsp. *tructae* DSM 21502^T^. These four strains lack lactose phosphotransferase and β-galactosidase, but *L*. *lactis* subsp. *tructae* DSM 21502^T^ and *L. fujiensis* JCM16395^T^ could utilize lactose in the API 50 test (Table 4). Therefore, we inferred that they have additional pathways for lactose metabolism. Ribokinase (EC 2.7.1.15) is the key enzyme in d-ribose metabolism. *L*. *raffinolactis* ATCC 43920^T^, *L*. *plantarum* DSM 20686^T^, *L*. *lactis* subsp. *hordniae* DSM 20450^T^, and *L*. *chungangensis* DSM 22330^T^ have no genes encoding ribokinase, so they cannot utilize d-ribose.

## Discussion

It is considerable realistic significance for applying *Lactococcus* strains to systematically analyze the evolutionary history and phylogenetic position of the *Lactococcus* species/subspecies. Therefore, we completed the whole-genome sequencing of ten type strains in *Lactococcus* genus to assess their genomic characteristics, metabolic diversity, and phylogenetic relationships.

Across ten *Lactococcus* species, the total genome size varied from 1.99 (*L*. *plantarum*) to 2.46 Mb (*L*. *lactis* subsp*. lactis*) (Table 2). The genome size of the *Lactococcus* species evaluated was relatively small compared with other LAB genera, such as *Enterococcus* (2.31–5.27 Mb) and *Lactobacillus* (1.23–4.91 Mb) [32]. A smaller genome size was suggested to indicate adaptations for reproductive efficiency or competitiveness in new environments [39]. The G + C content of 10 strains ranged from 34.81 (*L*. *lactis* subsp*. hordniae*) to 39.67% (*L*. *raffinolactis*). The G + C content of genome is influenced by selection and mutation involving multiple factors, including the symbiotic lifestyle, environment, nitrogen fixation ability, aerobiosis, and how the *pol*IIIa subunits are combined [40]. In addition, analysis of the trends in pan-genome size of *Lactococcus* species/subspecies proved that *Lactococcus* genus has an open pan-genome. This may be associated with the range of environments colonized by different *Lactococcus* species and the existence of numerous ways of exchanging genetic material. Previous reports showed that bacterial genomes change when they adapt to variable conditions, and that greater niche diversity requires larger pan-genomes [41].

Currently, the 16S rRNA gene sequencing technique and genomic DNA–DNA hybridization are considered the gold standards for identification of isolates at the species or genus level. However, because of conservative property, 16S rRNA gene could provide sufficient resolution at the species levels [42, 43], and DNA–DNA hybridization is rather time-consuming and labor-intensive. With advances in sequencing and computational technologies, the combination of core genome phylogeny and ANI values could provide accurate taxonomic guidance for LAB based on whole-genome sequences [44]. The lowest value for the *Lactococcus* genus was 67.4%, while the highest ANI value was 97.9%, revealing that the genomic sequence identities between different pairs of species had marked differences. The ANI values were mainly distributed in the region of 67–68% (Additional file 1: Table S1), while the distribution of *Bifidobacterium* genus was concentrated in the region of 71–74% [29]. These results suggest that the majority of sequence identities between *Lactococcus* genomes were low, only slightly higher than those between species from different genera. This could be used as a reference characteristic for identification of *Lactococcus* species.

Differences in carbohydrate metabolism genes among the ten *Lactococcus* species/subspecies showed that each could utilize various different carbohydrates. One interesting application of comparative genomics is the identification of associations between strains and their origins. Previous studies using comparative genomics in the genera *Nocardiopsis* [45] and *Bifidobacterium* [29] demonstrated specificity to particular ecological niches. In this study, the genotypic and phenotypic carbohydrate metabolism analyses suggested that the lactococcal species may correlate with respect to their origins, but regularities were not found. This is probably due to the unequal and small numbers of isolates from different origins. Three species were isolated from milk and four species from fish, bovine, and leafhopper, whereas only two species were isolated from plants and one species from activated sludge foam (Table 1). As more species are discovered from different countries and origins, further genomic evidence may become available for the genus *Lactococcus*, which would provide strong indications of the factors that have affected its evolutionary history. The majority of the carbohydrate fermentation results are in accordance with the metabolism pathway predicted by the genome sequence, but some differences were also detected, for which there are at least two explanations: predicted gene function based on sequence data is not completely accurate, and the pathways of carbohydrate metabolism are not yet well understood in KEGG.

## Conclusion

In the present study, ten type isolates of species/subspecies from the genus *Lactococcus* were sequenced and analyzed. Comparative genomic analysis revealed that species in the genus *Lactococcus* had relatively small and diverse genomes. The high degrees of diversity in genome size, gene content, and carbohydrate metabolism may be because the isolates originated from a wide range of host types and ecological niches. The genus *Lactococcus* has an open pan-genome, and thus the size of the pan-genome is as yet underestimated and will increase as additional isolates and species are sequenced. Since we had only a limited number of isolates, gene evolution in the genus *Lactococcus* was not analyzed. Nonetheless, this study provided insights into genomic and metabolic diversity and phylogenetic relationships of the majority of type species/subspecies in the genus *Lactococcus*, which will lay the foundation for future studies of their taxonomy and functional genomics.

## Additional files

Additional file 1: Table S1. Glycolysis/gluconeogenesis and the pentose phosphate pathway of ten type strains in the *Lactococcus* genus. The names of the genes that were present in the genomes of ten type strains in *Lactococcus* genus are shown in red, and those that were absent in all strains are shown in gray. (JPEG 336 kb)

Additional file 2: Figure S1. Pairwise ANI values across ten *Lactococcus* genomes. (DOC 42 kb)

## Abbreviations

ANI: Average nucleotide identity
CDS: Coding sequences
KEGG: Kyoto Encyclopedia of Genes and Genomes
*L.*: *Lactococcus*
LAB: Lactic acid bacterium
NCBI: National Center for Biotechnology Information
*S.*: *Streptococcus*
