# Supplementary material for: Genome-level comparisons provide insight into the phylogeny and metabolic diversity of species within the genus Lactococcus
Source: BMC Microbiol. 2017 Nov 3;17:213. doi: 10.1186/s12866-017-1120-5 (PMC5670709; doi:10.1186/s12866-017-1120-5)
Supplement: Supplementary file 1 — Glycolysis/gluconeogenesis and the pentose phosphate pathway of ten type strains in the Lactococcus genus. The names of the genes that were present in the genomes of ten type strains in Lactococcus genus are shown in red, and those that were absent in all strains are shown in gray. (JPEG 336 kb) [file 12866_2017_1120_MOESM1_ESM.doc]

***Table S1. Pairwise ANI values across ten Lactococcus genomes***

|  | DSM 22330 | DSM 20684 | JCM 16395 | ATCC 19257 | DSM 20450 | ATCC 19435 | DSM 21502 | DSM 6634 | DSM 20686 | ATCC 43920 |
| --- | --- | --- | --- | --- | --- | --- | --- | --- | --- | --- |
| *Lactococcus*  *chungangensis* DSM22330 | 100.00 |  |  |  |  |  |  |  |  |  |
| *Lactococcus* *garvieae* DSM20684 | 67.91 | 100.00 |  |  |  |  |  |  |  |  |
| *Lactococcus fujiensis* JCM16395 | 67.42 | 69.71 | 100.00 |  |  |  |  |  |  |  |
| *Lactococcus* *lactis* subsp. *cremoris* ATCC 19257 | 68.15 | 71.43 | 71.59 | 100.00 |  |  |  |  |  |  |
| *Lactococcus* *lactis* subsp. *hordniae* DSM 20450 | 68.16 | 71.34 | 71.85 | 86.17 | 100.00 |  |  |  |  |  |
| *Lactococcus* *lactis* subsp. *lactis* ATCC 19435 | 68.07 | 71.66 | 71.77 | 86.45 | 97.36 | 100.00 |  |  |  |  |
| *Lactococcus* *lactis* subsp. *tructae* DSM 21502 | 68.15 | 71.23 | 71.25 | 97.85 | 86.05 | 86.12 | 100.00 |  |  |  |
| *Lactococcus* *piscium* DSM 6634 | 75.81 | 67.79 | 67.37 | 68.67 | 67.92 | 68.12 | 68.07 | 100.00 |  |  |
| *Lactococcus* *plantarum* DSM 20686 | 75.44 | 67.91 | 67.30 | 68.21 | 68.32 | 68.58 | 68.34 | 76.88 | 100.00 |  |
| *Lactococcus* *raffinolactis* ATCC 43920 | 84.97 | 67.69 | 67.33 | 68.91 | 68.28 | 68.23 | 68.75 | 75.96 | 74.88 | 100.00 |
