# Supplementary figures and images for: Genome-level comparisons provide insight into the phylogeny and metabolic diversity of species within the genus Lactococcus
Source: BMC Microbiol. 2017 Nov 3;17:213. doi: 10.1186/s12866-017-1120-5 (PMC5670709; doi:10.1186/s12866-017-1120-5)

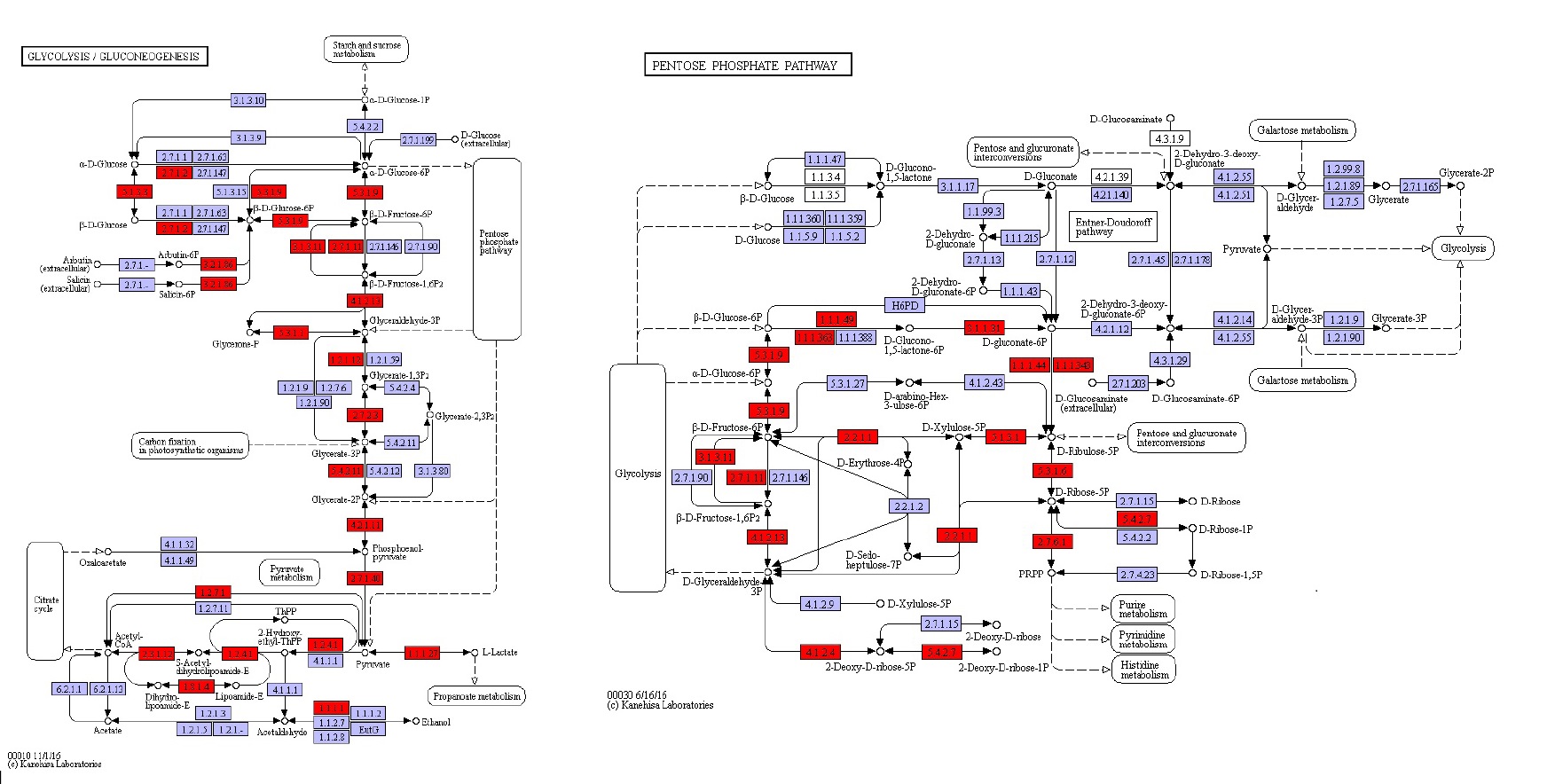

Supplement: Supplementary file 2 — Pairwise ANI values across ten Lactococcus genomes. (DOC 42 kb) [file 12866_2017_1120_MOESM2_ESM.jpg]
